# Omi inhibition ameliorates neuron apoptosis and neurological deficit after subarachnoid hemorrhage in rats

**DOI:** 10.1007/s13258-021-01176-y

**Published:** 2021-10-22

**Authors:** Yuanfeng Du, Dingbo Yang, Xiaoqiao Dong, Quan Du, Ding Wang, Yongfeng Shen, Wenhua Yu

**Affiliations:** Department of Neurosurgery, Nanjing Medical University Affiliated Hangzhou Hospital, Hangzhou First People’s Hospital, 261 Huansha Road, Hangzhou, 310000 China

**Keywords:** Protease Omi, Subarachnoid hemorrhage, Neurological deficit, Cell apoptosis, Early brain injury

## Abstract

**Background:**

Subarachnoid hemorrhage (SAH) is a severe neurological emergency, resulting in cognitive impairments and threatening human's health. Currently, SAH has no effective treatment. It is urgent to search for an effective therapy for SAH.

**Objective:**

To explore the expression of Omi protein after subarachnoid hemorrhage in rats.

**Methods:**

SAH rat model was established by injecting blood into the prechiasmatic cistern. Neurological deficit was assessed by detecting neurological deficit scores and brain tissue water contents. Apoptotic cells were evaluated by TUNEL staining and IHC staining. Omi and Cleaved caspase 3 expressions in nerve cells were determined by double staining using IF. Apoptosis-related proteins were measured by Western blotting assay.

**Results:**

SAH rat model was successfully established, showing more apoptotic cells and high neurological deficit scores in SAH rat. In SAH rat model, Omi expression in nerve cells was elevated and the upregulation of Omi mainly occurred in cytoplasm, accompanied by the degradation of XIAP and the increased cleaved caspase 3/9 and cleaved PARP. Once treated with UCF-101, a specific inhibitor of Omi, the increased cell apoptosis, left/right brain moisture contents and neurological deficits were notably reversed in SAH rat brain. Of note, SAH-induced the increases of apoptosis-related protein in nerve cells were also rescued by the administration of UCF-101.

**Conclusions:**

UCF-101-mediated Omi inhibition decreased the degradation of XIAP and subsequently inhibited the activation of apoptosis-related proteins, decreased nerve cell apoptosis, leading to the improvement on early brain injury in SAH rat. UCF-101-based Omi inhibition may be used to treat SAH with great potential application.

## Introduction

Subarachnoid hemorrhage (SAH) is a severe subtype of stroke affecting patients at a mean age of 55 years, accounting for 5% of all strokes, with a mortality rate of 33.3% and a disability rate of 16.7% (Macdonald and Schweizer 2017; Mejdoubi et al. 2018). Most patients die in one month after SAH, and two thirds of all deaths occur within the first 48 h after SAH (Dennis et al. 2001). Additionally, survivors often have cognitive impairments, which in turn affect patients’ daily functionality, labor capacity, and quality of life (Plata-Bello et al. 2017). Although many common therapies for SAH have created such as care at high-volume centers with dedicated neurointensive care units and surgical treatment (Dority and Oldham 2016), the management remains controversial and need further studies to clarify their role in improving patient outcome. Accumulating evidence indicates early brain injury is a leading cause of disability and death in patients with SAH (Chen et al. 2014; Sehba et al. 2012). In addition, alleviating early brain damage contributes to the improvement on survival rate and prognosis of SAH patients (Fujii et al. 2013; Zhang et al. 2017).

Early brain injury, which occurs within 3 days after SAH, is deemed to be the main reason accounting for the poor prognosis of patients with SAH (Burkhardt et al. 2017). Neuronal apoptosis is the hallmark in the early brain injury after SAH, contributing to the irreversible acute brain injury (Ostrowski et al. 2006; Shi et al. 2017). Mitochondrial regulation of apoptosis as a nerve center plays an important role in the process of apoptosis. Mitochondria impairment causes reactive oxygen species (ROS) over-production, ultimately triggering neuronal apoptotic death apoptosis via promoting mitochondrial membrane permeability and the release of apoptosis mitochondrial proteins including mitochondrial Cyt C and Bax (Duris et al. 2011). Omi/HtrA2 is a proapoptotic mitochondrial serine protease which involves in caspase-dependent cell apoptosis progress. Omi/HtrA2 precursor can translocate to the mitochondria, which is released to the cytosol after an apoptotic stimulation resulting in apoptosis (Hu et al. 2019; Vande Walle et al. 2008). Omi/HtrA2 binds to X-linked inhibitor of apoptosis protein (XIAP) and results in their displacement from activated caspases 9, thus activating caspase-dependent apoptosis (Russell et al. 2008). Higher level of Omi/HtrA2 distinctly promotes neuronal apoptosis in cerebral ischemia/reperfusion (Althaus et al. 2007), hippocampal neurons injury (Rami et al. 2010), and neurodegenerative diseases (Li et al. 2010), etc. Pre-treatment with UCF-101, a selective inhibitor of Omi/HtrA2 protease activity significantly reduces neuronal cell apoptosis and attenuates sepsis-induced cognitive dysfunction (Cilenti et al. 2003; Hu et al. 2013), protects against cerebral ischemia/reperfusion injury (Su et al. 2009), and traumatic spinal cord injury-induced locomotor impairments (Reigada et al. 2015). Therefore, the regulation of Omi/HtrA2 activity possibly facilitates the repair of neurological deficit and early brain injury via anti-apoptosis effect. However, whether protease Omi/HtrA2 is involved in apoptosis regulation of patients with SAH remains unclear.

Herein, we firstly explored the expression of Omi protein after subarachnoid hemorrhage in rats. Furthermore, the effects of Omi inhibition using UCF-101 on neuron apoptosis and neurological deficit in SAH rat model were also verified. Our findings provide an overview of current evidence for managing early brain injury after SAH via regulating mitochondrial serine protease.

## Materials and methods

### Animal model and treatment

All experimental procedures were approved by the Institutional Animal Care and Use Committee (IACUC) of Nanjing Medical University and were in accordance with the NIH Guidelines for the Use of Animals in Neuroscience Research. Adult male Sprague–Dawley rats weighting 250–300 g were purchased from Charles River Laboratories (Peking, China). All rats were housed in a controlled humidity and temperature (24 ± 0.5 °C) room with a 12 h light and dark cycle. All rats were raised with free access to water and food. Briefly, rats were anesthetized with isoflurane (2% oxygen, 300 ml/min) and subsequently placed in a stereotactic frame (SA301, Sansbio. Ltd., Nanjing, China). After disinfection, 1.0 cm scalp coronal incision was made and a 1.0 mm-diameter hole was drilled about 4.5 mm from the anterior fontanel. Under the stereotactic guidance, fresh autologous non-heparinized arterial blood (0.35 mL) from the femoral artery was injected aseptically into the hole (prechiasmatic cistern) in 20 s with a syringe pump in the SAH group, and allowed the syringe pump to remain for more than 2 min, preventing blood flowing backwards and the leakage of cerebrospinal fluid (Reigada et al. 2015). Rats in Sham group underwent the same procedure; however, the rats in the sham group were injected with 0.35 ml saline. The skin incision was sutured after removal of the suture. Subsequently, 2 ml saline was injected subcutaneously. All rats were allowed to recover in a head-down prone position for 45 min post-SAH at 30 °C. At the end of the operation procedures, the rats were housed in their corresponding cages in a controlled temperature (24 ± 0.5 °C). Sham control and model rats were sacrificed at 24 h (n = 10/each group), 48 h (n = 5/each group) and 72 h (n = 5/each group) after operation. For the treatment of UCF-101 (496,150, sigma), rats were subjected by intraperitoneal injection of 3.0 μmol/kg UCF-101 on 0.5 h before surgery and 12 h post-surgery in SAH rat. Rats in control group were injected the equal saline at the same time points. Consistently, rats were sacrificed at 24 h (n = 15/each group), 48 h (n = 10/each group) and 72 h (n = 10/each group) after operation. The whole brain and/or the left and right brain, cerebellum, brainstem in each group were harvested and stored at liquid nitrogen or immediately programmed for the subsequent experiments.

### Neurological deficit score

The scores were produced according to behavioural activity indicators including appetite, activity and neurological deficits (Zhang et al. 2016). For the appetite index, all rats were scored as follows: 0 points, finished meal; 1 point, left meal unfinished; 2 points, scarcely ate. For the activity index, all rats were scored as follows: 0 points, walk and reach at least three corners of the cage; 1 point, walk with some stimulation; 2 points, almost always lying down. For the neurological deficits index, all rats were scored as follows: 0 points, no deficits; 1 point, unstable walk; 2 points, impossible to walk. Neurological deficit scores were collected for each rat at 24 h after SAH based on the independent observations by three independent researchers who were blind to this study.

### Brain tissue water content

At 24 h after SAH model establishing, the left and right brain, cerebellum, brainstem were collected for detecting the wet weight. After allowing to dry for 24 h at 80 °C oven, the dry weight of the left and right brain, cerebellum, brainstem were measured. Then the brain tissue water content was calculated using the formula: (wet weight-dry weight)/wet weight*100%.

### TUNEL assay

At 24, 48, and 72 h after SAH, brain tissues of each group were collected and fixed in the 4% paraformaldehyde. After embedded by paraffin, the 10 μm-thickness specimens were dewaxed in xylene and rehydrated in gradient ethanol. Subsequently, the slices were incubated with DNase-free Protein K for 25 min at 37 °C and washed for thrice using PBS. After 20 min incubation of cell penetrating solution and three-times washing with PBS, TUNEL staining was performed using in situ Apoptosis Detection Kit (11,684,817,910, Roche, USA) according to the manufacture’s instructions for quantification of neuronal apoptosis. The number of TUNEL-positive neurons was counted manually in the ipsilateral cortex. Six sections per brain over a microscopic field of 20 × were averaged. Data were presented as the ratio of TUNEL-positive neurons (%). Then the slices were stained by DAPI for 10 min in darkness. After mounting, the slices were photographed by fluorescence microscope. The number of apoptotic cells were analysed by using Image Pro Plus software.

### Immunofluorescence

Brain tissues were collected at 24 h, 48 h, 72 h after SAH, fixed in the 4% paraformaldehyde and embedded by paraffin. After deparaffinization in xylene and hydration in gradient ethanol, the slices were incubated with antigen retrieval solution, followed by the incubation of immunofluorescence quenching agents to block the intrinsic fluorescence. Subsequently, the sections were blocked by BSA solution and incubated with antibodies against NeuN (1:100, CST, Ma, US), Omi (1:200, Abcam, China) and cleaved Caspase 3 (1:200, affinity, China) at 4 °C overnight. Then, the sections were washed with PBS and incubated with fluorescence-conjugated secondary antibodies Goat Anti-Rabbit IgG (H + L) FITC-conjugated (1:200, affinity, China) and/or Goat Anti-Mouse IgG (H + L) CY3-conjugated (1:200, affinity, China) for 1 h at room temperature. After staining with DAPI, the slices were mounted and photographed by fluorescence microscope.

### Western blotting

Total protein was extracted from brain tissues, was obtained at 24 h after SAH, using RIPA solution (Beyotime, Shanghai, China). Cytoplasmic and mitochondrial proteins were extracted according to the manufacture’s instruction (C7610, Solarbio, Beijing). In brief, brain tissues were cut into pieces on the ice and the tissue pieces resuspended in cytoplasmic extraction buffer were homogenized using homogenizer. The homogenate was collected and centrifugated for 10 min at 3000 rpm at 4 °C. The supernatants were centrifugated again for 30 min at 12,000 rpm at 4 °C, and subsequently the supernatants were collected and stored as the cytoplasmic protein. Then, the pellets were applied to extract mitochondrial protein. Briefly, the pellets were lysed using 0.1 ml cytoplasmic extraction buffer. After centrifugation, the precipitates were lysed with 0.1 ml mitochondrial extraction buffer for 30 on the ice. Subsequently, the mixture was centrifugated for 10 min at 13,000 rpm at 4 °C and the supernatants were harvested as the mitochondrial protein. Protein concentration was assessed by BCA kits. 30 μg protein was performed SDS–polyacrylamide gel electrophoresis (SDS-PAGE) and transferred to the activated polyvinylidene difluoride (PVDF). After blocking with 5% non-fat blocking grade milk (Bio-Rad, Hercules, CA, USA), the membrane was incubated with the following primary antibodies overnight at 4 °C (anti-Cleaved Caspase3 (1:2000, Abcam, China), anti-Cleaved Caspase9 (1:1000, Cell Signaling Technology Inc., MA, USA), anti-XIAP (1:2000, Abcam, China), anti-PARP (1:2000, Abcam, China), anti-Omi (1:2000, Abcam, China), anti-VDAC1 (1:1000, Affinity, China) and anti-GAPDH (1:1000, Cell Signaling Technology Inc., MA, USA)). On the following day, the membranes were incubated with the corresponding HRP-labeled secondary antibody (1:2000, Abcam, China) at room temperature for 1 h. Immunoblots were then visualized with ECL chemiluminescence reagent kit (Millipore, Billerica, MA, USA) and quantified with optical methods using the ImageJ software (ImageJ 1.5, NIH, USA). The results were normalized using GAPDH as an internal control.

### Immunohistochemistry

Brain tissue were from rat at 24 h, 48 h, and 72 h after SAH. Consistently, 10 μm-thickness paraffin sections were dewaxed in xylene, rehydrated in gradient ethanol and processed antigen retrieval using high pressure method. After blocking using 3% H_2_O_2_ and 3% BSA, the sections were incubated with primary antibodies against Cleaved Caspase3 (1:2000, abcam, China) and Cleaved Caspase9 (1:2000, abcam, China) at 4 °C overnight. Subsequently, all slices were incubated with secondary antibody labelled with streptavidin-HRP (1:2000, Abcam, China) at room temperature for 50 min. After stained with DAB staining buffer, the slices were re-stained by hematoxylin staining for 3 min. After mounting using neutral balsam, the staining results were visualized with the microscope (Olympus BX41).

### Statistical analysis

Data were presented as the mean ± standard deviation (SD) and analysed using GraphPad Prism 7 (La Jolla, CA, USA). Positive cells in IF and IHC assays were analysed by using Image Pro Plus 6.0 software. Statistical evaluation of the data was performed by Student’s t-test’ or one-way ANOVA followed by Least Significant Difference (LSD) analysis of variance. A p-value < 0.05 was considered statistically significant.

## Results

### SAH model is successfully established

**Fig. 1:**
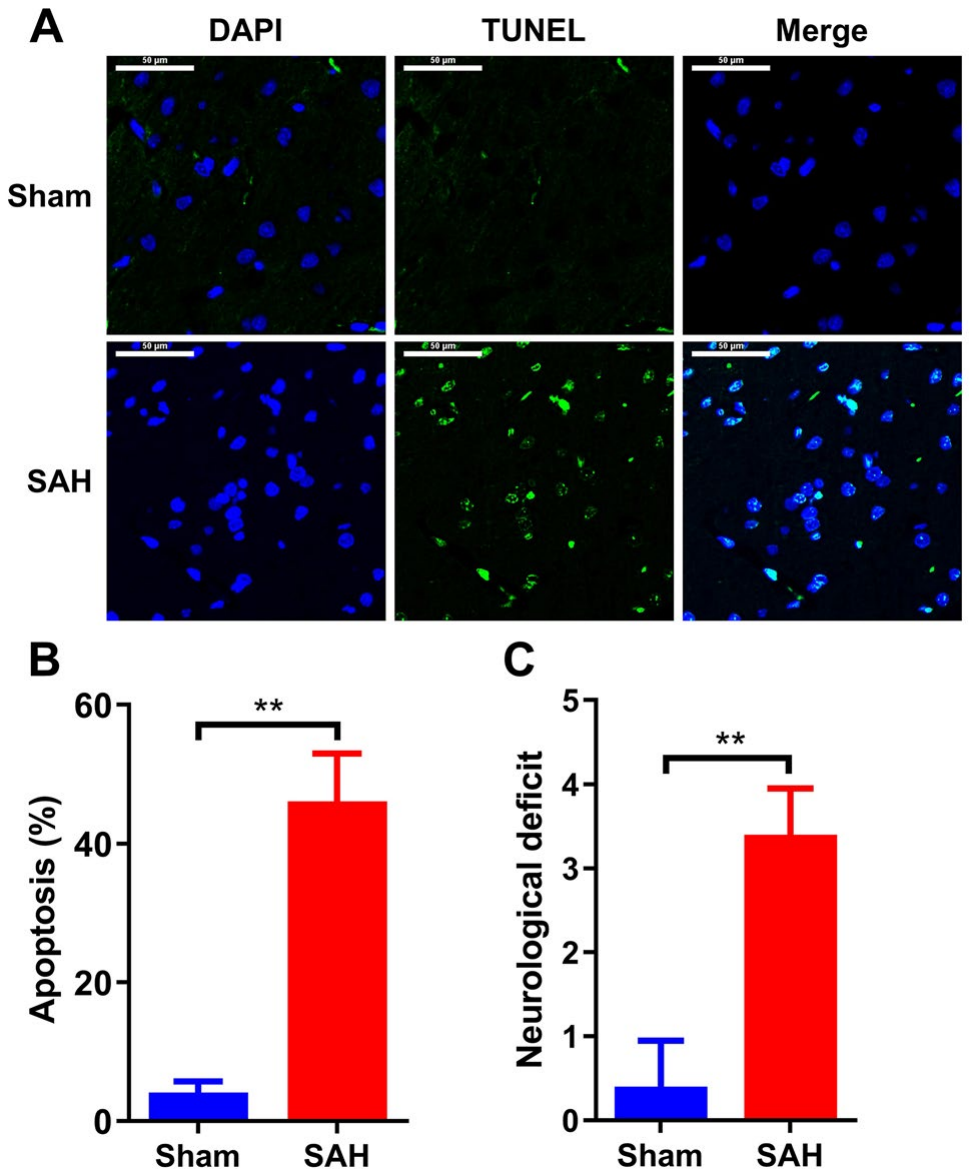
Model establishing of SAH. **A** All rats were randomly divided into two groups: Sham group and SAH group (n = 20/each group). Brain tissues were collected at 24 h after SAH in rats. Then apoptotic cells were determined by using TUNEL assay (n = 5/each group). **B** Analysis of apoptotic cells in the brain tissues of panel (**A**). **C** Neurological deficit scores of normal rats and SAH rats. Scale bar: 50 μm. **p < 0.01

SAH rat model was established by injecting blood into the prechiasmatic cistern. Early brain injury is common occurred at 24 h after SAH in rats (Shen et al. 2020). To verify whether SAH model was successfully established, brain tissues were harvested firstly at 24 h after SAH. In the TUNEL staining results, robust elevated apoptotic cells were observed in SAH rat compared to that in the Sham group (Fig. 1A), increasing from 3 to 45% of the number of apoptotic cells in SAH model (Fig. 1B). Additionally, the increase of neurological deficit score was also verified in SAH rat compared to the control rat, increased by approximately sevenfold (Fig. 1C). Therefore, the model of SAH and subsequent early brain injury was successfully established.

### Omi expression is elevated in nerve cells of SAH rats

**Fig. 2:**
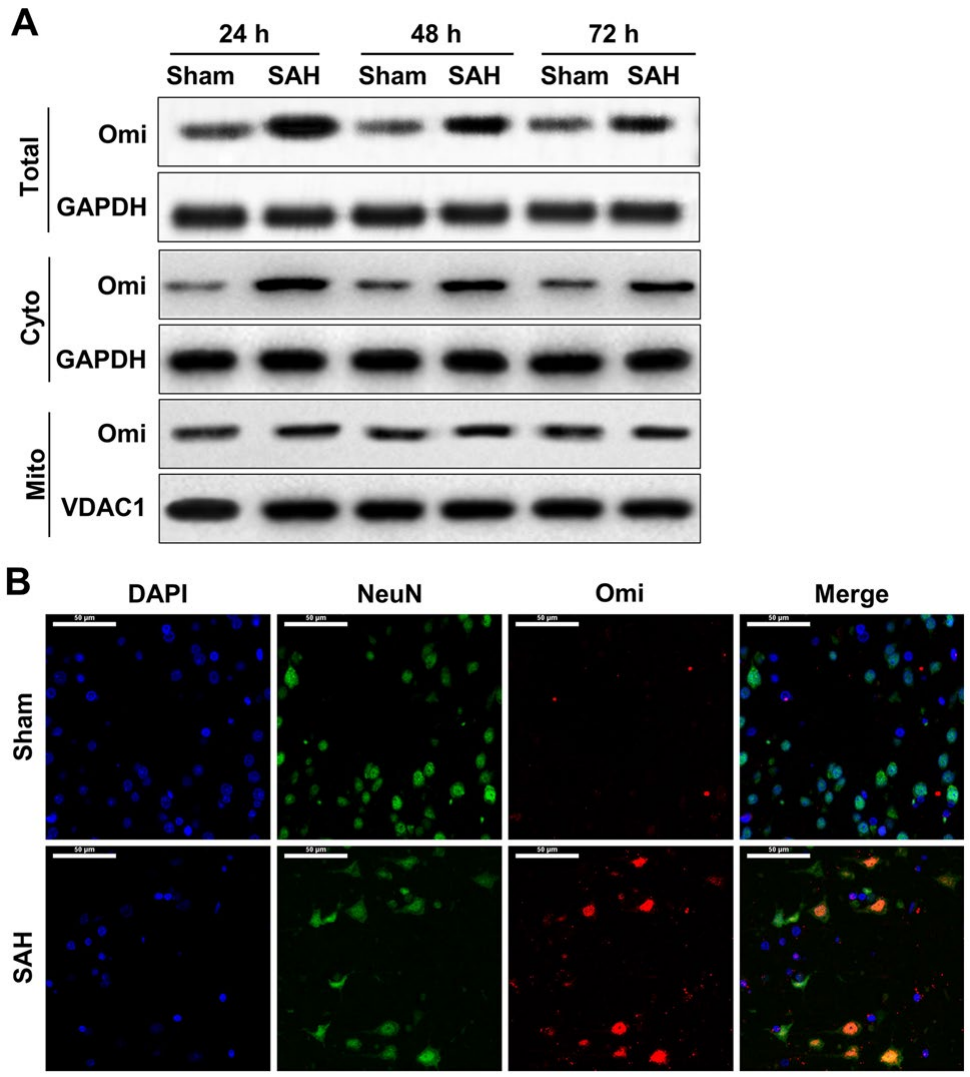
Omi expression in nerve cells in SAH rats. **A** At 24 (n = 5/each group), 48 (n = 5/each group), and 72 h (n = 5/each group) after SAH, rat brain tissues were collected and total protein, cytoplasmic protein and mitochondrial protein were extracted from brain tissues of Sham control and SAH rats. Western blotting assay was used to measure protein expression of Omi in total brain tissue, cytoplasmic and mitochondrial proteins. **B** After the brain tissue obtained from SAH rats at 24 h, double staining of neuron marker NeuN and Omi were performed (n = 5/each group). Green: NeuN-positive cells; Red: Omi-positive cells. Scale bar: 50 μm (colour figure online)

To investigate whether protease Omi/HtrA2 is involved in the progression of SAH, the expression of Omi in brain tissues of SAH rat was firstly determined. In the brain tissues of SAH model, either at 24 h and 48 h or 72 h after SAH, the protein expression of Omi increased significantly compared to its level in brain tissue from rats in Sham group, showing the highest level at 24 h after SAH (Fig. 2A). It is reported that Omi play cellular regulation role relying on its release from mitochondria to cytoplasm. Subsequently, cytoplasmic and mitochondrial Omi levels were also assessed in brain tissue of SAH rats. As shown in Fig. 2, the upregulation of Omi protein in brain tissues of SAH rats mainly concentrated in cytoplasm (Fig. 2A), which implied that the release of Omi from mitochondria to cytoplasm was observed in SAH rats. On the following experiments, the expression of Omi in nerve cells was also detected in the two group rats. As expected, in nerve cells that was labeled with NeuN antibody (green), fluorescence intensity of Omi (red) was also notably elevated in SAH rat, which was almost no Omi-positive nerve cells in rat brain of Sham group (Fig. 2B). These data demonstrated an enhancement of Omi level in rat brain after SAH, which possibly mainly occurred in the nerve cells.

### XIAP degradation and subsequent cell apoptosis occur in rat brain after SAH

**Fig. 3:**
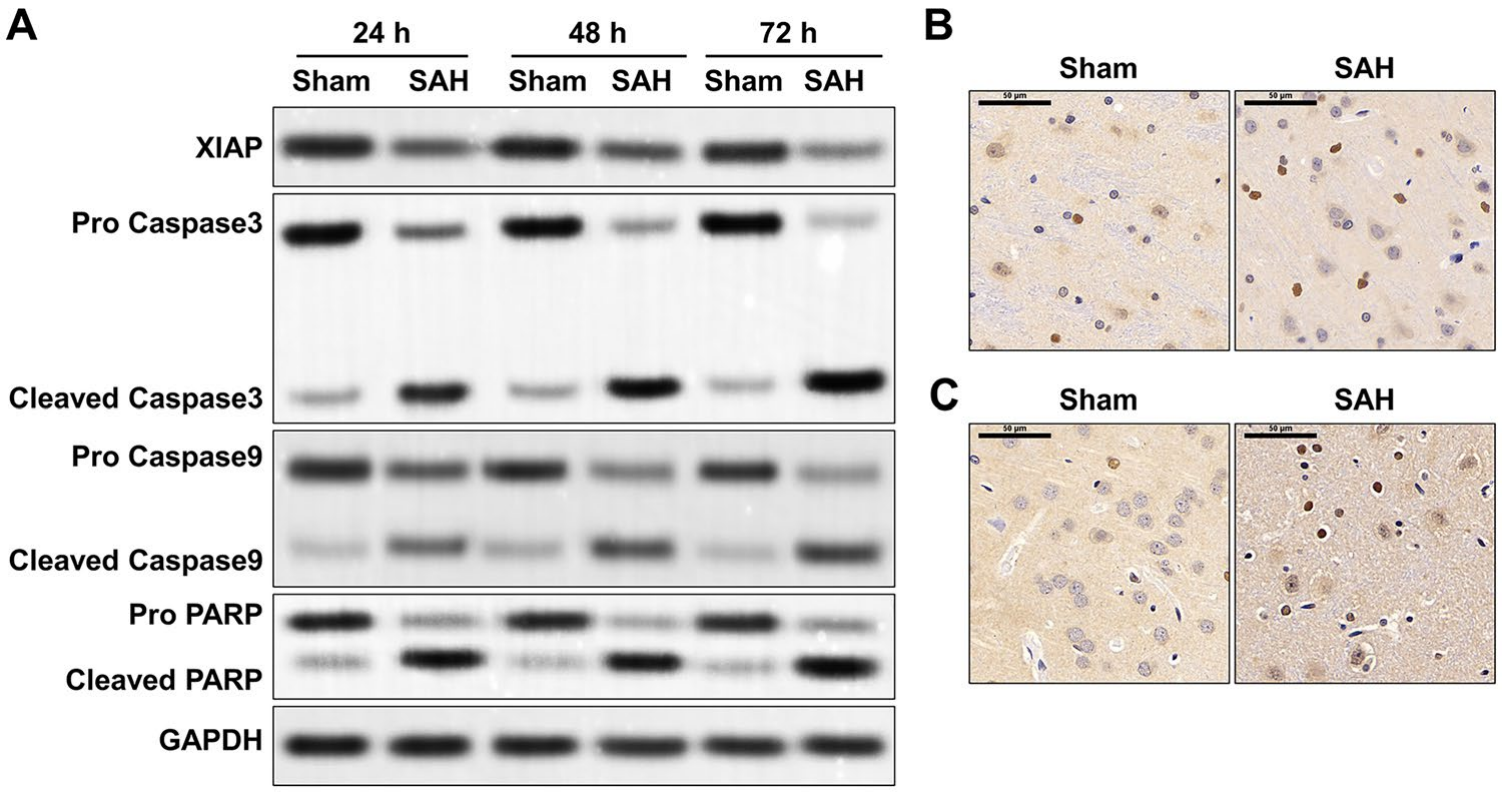
Expression of XIAP and apoptosis-related events in SAH rat. **A** In sham and SAH groups, total protein of brain tissues were extracted at 24 h (n = 5/each group), 48 h (n = 5/each group), and 72 h (n = 5/each group) after SAH. Then protein levels of XIAP, cleaved caspase 3/9, Pro caspase 3/9, cleaved PARP and Pro PARP were determined by Western blotting assay. At 24 h (n = 5/each group) after SAH, the IHC staining of cleaved caspase 3 (**B**) and cleaved caspase 9 (**C**) were performed in the brain tissue from Sham and SAH groups. Scale bar: 50 μm

Next, the association between increased Omi and increased apoptotic cells were explored. At 24, 48 and 72 h after SAH, the expression of XIAP robustly declined in brain tissues of SAH rats, and the degradation rate reached the highest at 48 h after SAH (Fig. 3A). Along with the decrease of XIAP, apoptosis-related events including cleaved caspase 3/9, and cleaved PARP were obviously activated, accompanied by the decline in the full length of pro caspase 3/9 and pro PARP, in SAH rat compared to that in the Sham group (Fig. 3A). Additionally, based on the IHC staining, more cleaved caspase 3- and cleaved caspase 9-positive cells were observed in rat brain tissues at 24 h after SAH (Fig. 3B, C). It has confirmed that XIAP degradation facilitates the activation of caspase 3/9 and PARP (Goo et al. 2014). These findings suggested apoptosis-related proteins were increased in SAH model, which might be due to the degradation of XIAP.

## Omi inhibition reverses neuron apoptosis and neurological deficit in SAH rat

**Fig. 4:**
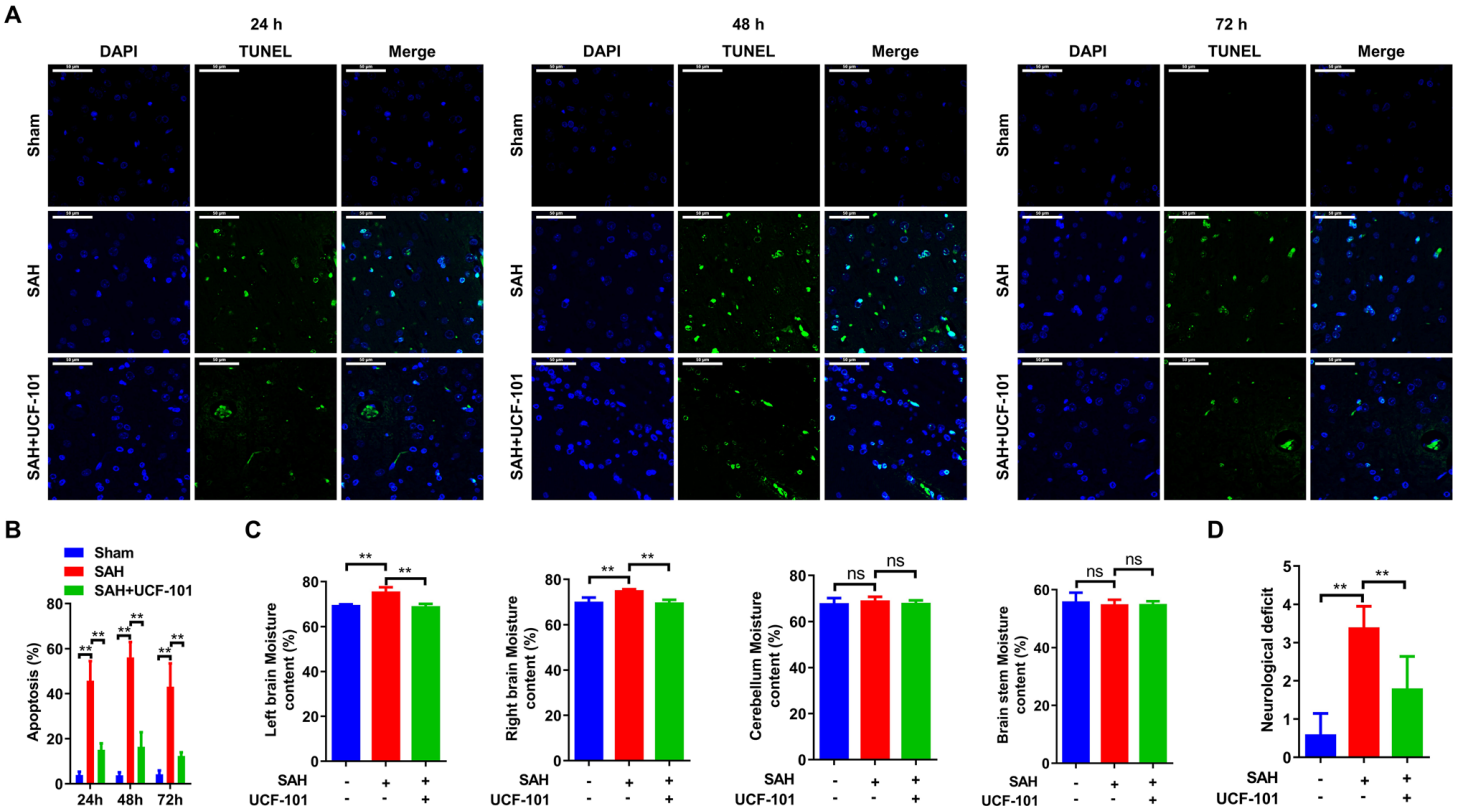
Effects of UCF-101 on neuron apoptosis and neurological deficit after subarachnoid hemorrhage in rats. **A** All rats were divided into three groups: Sham group (replaced with saline injection into the prechiasmatic cistern, n = 35), SAH model group (fresh autologous non-heparinized arterial blood injection into the prechiasmatic cistern and intraperitoneal injection of PBS, n = 35), and SAH + UCF-101 group (fresh autologous non-heparinized arterial blood injection into the prechiasmatic cistern and intraperitoneal injection of UCF-101, n = 35). After modeling for 24, 48, and 72 h, brain tissues were harvested and performed by TUNEL staining (n = 5/each group at each time point). Green: apoptotic cells. **B** Analysis of apoptotic cells in the brain tissues of panel (**A**). **C** After weighting the wet and the dry weight of left and right brain, cerebellum, brainstem were measured, the brain tissue water content was contrastively analysed (n = 5/each group). **D** Neurological deficit scores of normal rats, SAH rats and SAH rats exposed to UCF-101. **p < 0.01. ns: no significant. Scale bar: 50 μm (colour figure online)

In rat brain, more apoptotic cells were verified at 48 h after SAH compared to that at 24 h or 72 h (Fig. 4A, B). Once treated with UCF-101, a specific inhibitor of Omi, the number and the proportion of apoptotic cells were significantly decreased at 24 h, 48 h and 72 h after SAH in comparison to that in SAH rats (Fig. 4A, B). Next, neurological function was also determined in the three group rats. As shown in Fig. 4C, the left/right brain moisture contents were significantly promoted in SAH rat, while UCF-101 administration almost reversed these changes to normalized level (Fig. 4C). By contrast, the moisture contents in cerebellum and brainstem showed no obvious changes, no matter in SAH model, but also in SAH rats treated with UCF-101 (Fig. 4C). Furthermore, a declined neurological deficit scores was verified in UCF-101-exposed SAH rats compared to the PBS-exposed SAH rats (Fig. 4D). Possibly, UCF-101-mediated the inactivation of Omi could reverse SAH-induced early brain injury via inhibiting cell apoptosis.

### Omi inhibition abolishes SAH-induced the cleavage of apoptosis-related protein

**Fig. 5:**
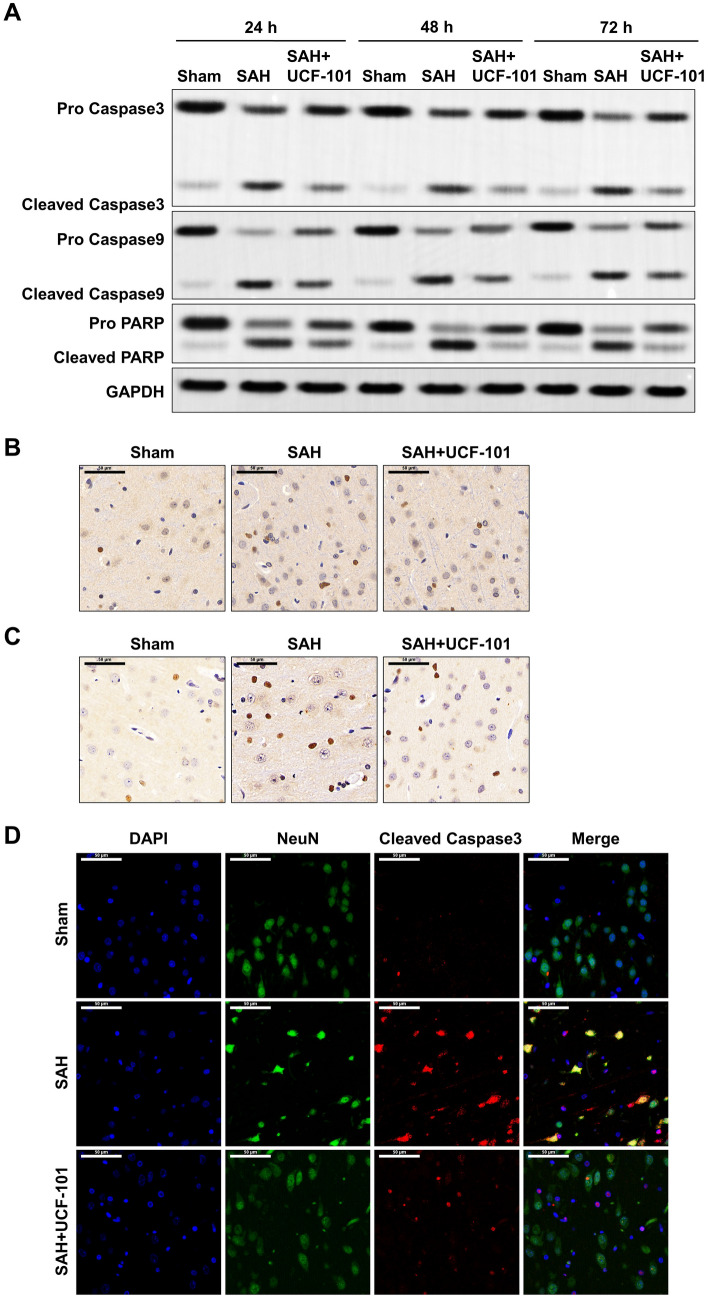
The alterations of apoptosis-related proteins in SAH model treated with UCF-101. **A** In brain tissues from Sham, SAH and SAH + UCF-101 groups, protein levels of cleaved caspase 3/9, Pro caspase 3/9, cleaved PARP and Pro PARP were determined by Western blotting assay (n = 5/each group at each time point). Brain tissues were harvested from the above three groups at 24 h after SAH, the expression of cleaved caspase 3 (**B**) and cleaved caspase 9 (**C**) were measured by IHC staining (n = 5/each group). **D** In the brain tissues of the above three groups, double staining of neuron marker NeuN and cleaved caspase 3 were performed using IF staining. Green: NeuN-positive cells; Red: cleaved caspase 3-positive cells. Scale bar: 50 μm (colour figure online)

As shown in Fig. 5A, in addition to the number of apoptotic cells, SAH-induced the increases of cleaved caspase 3/9 and PARP at 24 h, 48 h, and 72 h were significantly abolished when SAH rats were administrated with UCF-101 (Fig. 5A). In rat brain at 24 h after SAH, the number of cleaved caspase 3- and cleaved caspase 9-positive cells was also reversed by the administration of UCF-101 (Fig. 5B, C). Combined with the double staining with NeuN and cleaved caspase 3, SAH induced a great number of apoptotic nerve cells (NeuN- and cleaved caspase 3-positive cells) (Fig. 5D). However, the exposure of UCF-101 significantly reduced SAH-mediated nerve cell apoptosis at 24 h after SAH in rats (Fig. 5D). Collectively, UCF-1 mediated the inactivation of caspase 3/9 and PARP, resulting in inhibition of nerve cell apoptosis in SAH rats.

## Discussion

The management of neuronal apoptosis contributes to the treatment of early brain injury in patients with SAH (Mo et al. 2019). As an important mitochondrial protein, Omi disorder commonly induces neurological diseases (Lindholm et al. 2004), but rarely report indicates the association between Omi and SAH. In this study, UCF-101-mediated the inactivation of Omi notably reversed neuronal apoptosis and subsequently the increased brain tissue water content after SAH in rats. Thus, our data identified Omi might be a novel drug target for the prevention and treatment of SAH-related early brain injury.

Widespread accumulation of Omi/HtrA2 occurs in pathologic alpha-synuclein in brains with Parkinson disease (PD) (Kawamoto et al. 2008). Focal cerebral ischemia/reperfusion induces an up-regulation of mitochondrial Omi/HtrA2, resulting in an increased cytosolic accumulation of Omi/HtrA2 (Althaus et al. 2007). In the model of SAH, the accumulation of Omi in nerve cell was also observed along with the neurological injury. The data confirms that the activity of protease Omi/HtrA2 may be induced in brain tissue after SAH. The binding of Omi to XIAP competitively inhibited the inhibition effect of XIAP on the cleavage of Caspase protein, leading to the increased apoptotic cells (Goo et al. 2014). Conditional oxidative stress results in the translocation of Omi/HtrA2 from mitochondria to the cytosol, accompanied by the XIAP degradation and the elevation of cleaved caspase-3, caspase-9, and PARP levels, which is prevented by the pretreatment of UCF-101 (Wang et al. 2018). Our results demonstrated a significant decrease of XIAP, and a significant increase of cleaved apoptosis-related proteins, along with the elevation of cytoplasmic Omi expression in SAH rats. And Omi inhibition using UCF-101 abrogated SAH-launched nerve cell apoptosis. Therefore, the enhancement of Omi in brain tissue of rats with SAH promote neuronal apoptosis partly via integrating XIAP. Possibly, mitochondrial Omi released into cytoplasm, subsequently activated XIAP-caspases signaling pathway, leading to neuron apoptosis and neurological deficit in SAH rats. Higher expression of mitochondrial Omi/HtrA2 promotes cardiomyocyte apoptosis, not involved its translocation from the mitochondria, which may be through negatively regulating anti-apoptotic mitochondrial protein HAX-1 (Wang et al. 2016). Possibly, protease Omi/HtrA2-induced nerve cell apoptosis not least rests on XIAP signaling cascade, but also HAX-1 and others.

Conversely, a transgenic mouse model with neuron-specific overexpression of Omi/HtrA2 does not show any sign of apoptotic cell death, however, exhibits a protective role in neurons (Liu et al. 2007). Expression of Omi counteracted α-synuclein-induced neurotoxicity, showing a neuroprotective function (Chung et al. 2020). Mechanistically, Omi/HtrA2 regulates mitochondrial biogenesis and mitochondrial stress, participating in the progression of neuroprotection (Jeyaraju et al. 2009; Xu et al. 2014). In addition, abnormal accumulations of HtrA2/Omi exhibit in several types of motor neuronal inclusions, associating with the pathogenesis of amyotrophic lateral sclerosis (Kawamoto et al. 2010). Protease Omi facilitates neurite outgrowth by cleaving the transcription factor E2F1 in neuroblastoma cells, which is abolished by pretreatment with the specific Omi inhibitor UCF-101 (Ma et al. 2015). In the present study, Omi was elevated in the nerve cells of brain tissues from SAH rats, accompanied by the increase of brain tissue water contents and neurological deficit scores. Targeting regulation of HtrA2/Omi using UCF-101, the neurological deficit had comparatively obvious recovery. Thus, Omi showed a detrimental role in neuronal cells in response to SAH, which was different from its role in other types of neurological diseases, implying multiple functions of Omi in nerve-related diseases.

Abnormal autophagy facilitates caspase-dependent neuronal apoptosis (Chung et al. 2018). Enhancement of autophagy ameliorates neuronal apoptosis and subsequent neurological deficits after SAH in rats (Shao et al. 2016; Sun et al. 2019). By contrast, mitophagy-induced oxidative stress promotes neuronal death after SAH (Zhang et al. 2019). Appropriate activity of autophagy-lysosomal pathway exhibits a pro-survival mechanism in SAH, while excessive self-digestion leads to cell apoptosis after SAH (Wu et al. 2016). Protease Omi/HtrA2 is also involved in the autophagy cellular process. Omi/HtrA2 facilitates the degradation of mutant proteins involved in neurodegenerative diseases via autophagy regulation (Li et al. 2010). In SAH model, Omi inhibition declined caspase-dependent neuronal apoptosis. Whether Omi/HtrA2-mediated autophagy or mitophagy participates in the apoptosis regulation of neuronal cells after SAH needs further studies to verify.

## Conclusions

In summary, high expression of Omi was observed in nerve cells after SAH in rats, implying it as a promotor during SAH progression. Downregulation of Omi robustly ameliorated neuronal apoptosis, the decreased nerve cells, the increased brain tissue water contents and neurological deficit scores, resulting in the repair of neurological deficits in SAH rats. Probably, Omi may be a novel therapeutic treatment target in early brain injury of SAH and the specific inhibitor of Omi protease may have important application potential in SAH therapy.

## Data Availability

All data generated or analyzed during this study are included in this published article and its additional files.
